# Swelling of Homogeneous Alginate Gels with Multi-Stimuli Sensitivity

**DOI:** 10.3390/ijms24065064

**Published:** 2023-03-07

**Authors:** Haniyeh Malektaj, Aleksey D. Drozdov, Jesper deClaville Christiansen

**Affiliations:** Department of Materials and Production, Aalborg University, Fibigerstraede 16, 9220 Aalborg, Denmark

**Keywords:** alginate gel, ionic bonds, swelling, degradation, environmental sensitivity, catch bonds

## Abstract

A new two-step method is suggested for the preparation of homogeneous alginate gels. In the first step, alginate chains are weakly bonded by Ca${}^{2+}$ ions in an aqueous solution with a low pH. In the next step, the gel is immersed into a strong solution of CaCl${}_{2}$ to finalize the cross-linking process. Homogeneous alginate gels preserve their integrity in aqueous solutions with a pH ranging from 2 to 7 and ionic strength in the interval from 0 to 0.2 M, at temperatures ranging from room temperature up to 50 °C, and can be used in biomedical applications. The immersion of these gels into aqueous solutions with low pH induces the partial breakage of ionic bonds between chains (treated as gel degradation). This degradation affects the equilibrium and transient swelling of homogeneous alginate gels and makes them sensitive to the history of loading and environmental conditions (pH, ionic strength and temperature of aqueous solutions). As sensitivity to the environmental stimuli is a characteristic feature of polymer networks connected by catch bonds, homogeneous alginate gels may serve as a simple model, mimicking the behavior of more sophisticated structures in living matter.

## 1. Introduction

Hydrogels are networks of hydrophilic polymer chains connected by covalent and/or physical bonds. Due to the presence of permanent cross-links between chains, gels preserve their integrity when they are swollen in water.

Alginate is a linear polysaccharide composed of irregular blocks of β-D-mannuronic acid (M) and α-L-guluronic residues (G) [1,2]. To form a gel, sodium-alginate chains are ionically cross-linked (by the substitution of sodium ions with divalent cations that can bond the G-blocks [3]). The “egg-box” model (Figure S1) is traditionally adopted to describe the cross-linking process [4,5]. Due to their non-toxicity, bio-compatibility and degradability, alginate-based gels have found numerous applications in biomedicine (drug and protein delivery, wound dressing, scaffolds for cell growth and organoid morphogenesis, 3D bioprinting for tissue regeneration) [6,7,8,9,10], as well as in flexible electronics and environmental technologies [11].

The preparation of alginate gels using the standard method (mixture of solutions of alginate chains and calcium chloride CaCl${}_{2}$ [3]) results in rapid and poorly controlled gelation, the formation of heterogeneities, and, as a consequence, the strong deterioration of gels’ mechanical properties [12,13,14].

Two techniques are conventionally applied in the manufacture of macro- and micro-gels with ionic bonds formed by Ca${}^{2+}$ ions between alginate chains. In methods based on external Ca${}^{2+}$ sources, a pre-gel solution of alginate chains is immersed into an aqueous solution of a calcium salt. Ca${}^{2+}$ ions diffuse into the gel from the surrounding solution, forming ionic bonds with the guluronic acid residues. In approaches based on internal Ca${}^{2+}$ sources, alginate chains are mixed with the dispersion of an insoluble calcium salt (for example, CaCO${}_{3}$ or CaSO${}_{4}$), and Ca${}^{2+}$ ions are produced inside the gel by conversion of the insoluble salt into soluble salt using chemical reactions [15,16]. As both strategies are based on the diffusion (either Ca${}^{2+}$ cations or chelating agents necessary for the conversion of insoluble salts into soluble), they require modifications to ensure the uniform distribution of ionic cross-links [17,18]. Reference [19] suggested inducing the controlled release of Ca${}^{2+}$ ions from CaCO${}_{3}$ by the addition of glucono-δ-lactone, whose hydrolysis caused a reduction in the pH of the mixture and subsequent dissolution of CaCO${}_{3}$. The release of Ca${}^{2+}$ ions from CaCO${}_{3}$ via water electrolysis was proposed in [20]. The use of photoacid generators for the light-induced release of Ca${}^{2+}$ ions from CaCO${}_{3}$ was introduced in [21]. The replacement of deionized water in the preparation procedure with weak NaCl solutions was suggested in [22]. The simultaneous use of two types of divalent cations (Ca${}^{2+}$ and Ba${}^{2+}$) to cross-link alginate chains was recommended in [23]. A preparation procedure in which a pre-gel formed in an aqueous solution was immersed into an organic solvent, where it was cross-linked in the collapsed state, was proposed in [24]. A two-stage strategy (pre-crosslinking with internal sources and post-crosslinking with external sources of Ca${}^{2+}$ ions) for the 3D printing of alginate hydrogels was suggested in [25].

The objective of this study is twofold. Our first aim is to develop a facile method for the preparation of homogeneously cross-linked alginate gels and to investigate the effect of pH on their equilibrium and transient swelling. We focus on the pH-responsiveness of the gels, because this is of particular importance for the biomedical applications of alginate-based gels in targeted drug delivery (due to this property, the encapsulation of hydrophobic drugs and proteins is enhanced, physiological and pathological sites are distinguished under controlled release, and the concentration of hydrophobic drugs in plasma grows compared with the oral administration of their suspensions [26,27,28,29]) and wound healing (pH-triggered degradation of gels intensifies the release of antibiotics, combats bacterial infection and enhances therapeutic effects for acute infection [30,31,32]).

Conventional (covalent and physical) bonds between chains weaken under loading and remain insensitive to environmental conditions. The bonds that strengthen under deformation are conventionally referred to as “catch” bonds [33]. The strengthening of bonds in living tissues (the phenomenon known as “the strength on demand” [34]) is explained by mechanochemical transformations (the formation and dissociation of protein–ligand complexes) in biological materials [35,36]. Due to the complicated nature of mechanochemical processes governing receptor–ligand interactions, attention was recently paid to the development of synthetic materials mimicking the catch bond dynamics [37,38,39,40].

The other aim of this work is to show that uniform alginate gels demonstrate several important features of the catch bonds (in particular, a strong dependence on the environmental conditions). For this purpose, these gels (swollen at pH = 7) are immersed in aqueous solutions with pH = 2, and, afterwards, they are re-swollen in solutions with various pHs (ranging from 2 to 7) and ionic strengths (typical for biomedical applications) at temperatures *T* ranging from room temperature up to 50 °C. It is shown that the equilibrium degree of swelling and the kinetics of the re-swelling of these gels are noticeably affected by the loading history (the duration of degradation in aqueous solutions with pH = 2) and environmental factors (pH, ionic strength and temperature of aqueous solutions).

## 2. Materials and Methods

### 2.1. Materials

Alginic acid sodium salt from brown algae was purchased from Acros Organics (Geel, Belgium). Calcium chloride (CaCl${}_{2}$) and potassium hydroxide (KOH) were purchased from Sigma-Aldrich. Sodium chloride (NaCl) was provided by ThGeyer (Ballerup, Denmark), and 37% (*v*/*v*) hydrochloric acid (HCl) was supplied by VWR International (Rosny-sous-Bois, France). Deionized water was used in hydrogel preparation and measurements.

### 2.2. Preparation of Hydrogels

For comparison, three series of ionically cross-linked alginate gels were prepared.

**Figure 1:**
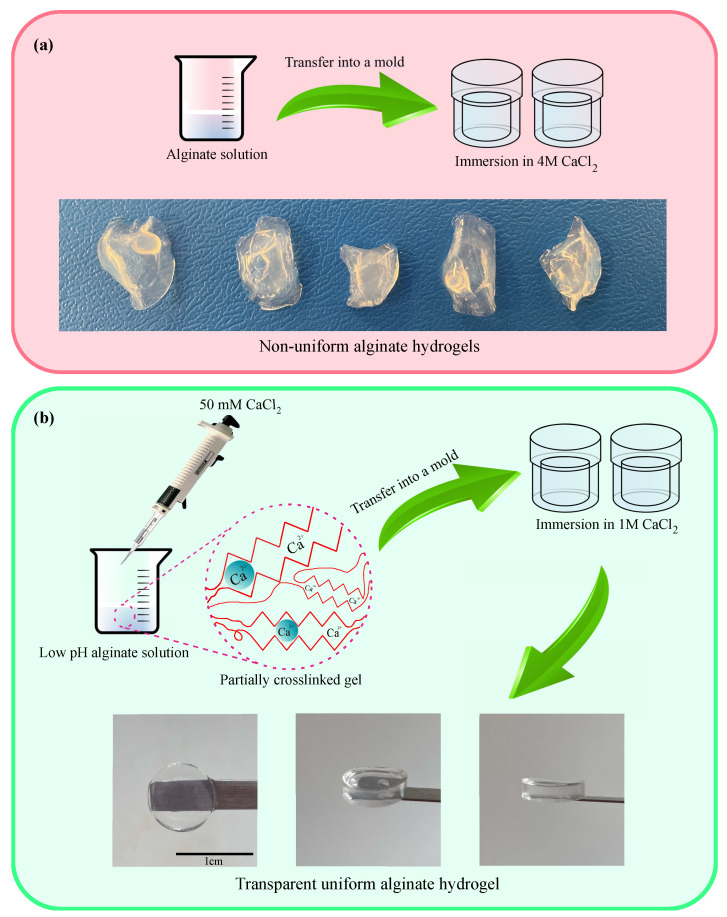
Preparation of alginate gels (**a**) by the conventional method, (**b**) by the method proposed in this work.

Hydrogels of the first series were prepared by the conventional method (which leads to the rapid gelation and formation of inhomogeneities) [1]. Alginate was dissolved in deionized water with a concentration of 1 wt%. The solution was poured into a mold (with thickness of 2 mm), and a small amount of 4 M CaCl${}_{2}$ solution was added to start gelation. After 10 min, the gel was separated from the mold and immersed into 25 mL of the 4 M CaCl${}_{2}$ solution for 1 day to finish the gelation process. Since the gelation occurred rapidly, the gel had an irregular shape and nonuniform structure (Figure 1a).

Hydrogels of the other series were prepared by a method that ensured that Ca${}^{2+}$ ions were uniformly dispersed. For this purpose, the pH of the alginate solution (1 wt%) was adjusted to 3.5 by adding HCl. Changes in pH were needed to reduce the ionization of carboxyl groups at the alginate backbone. The solution was mixed with 50 mM of CaCl${}_{2}$ solution in proportion 28:1 (*v*/*v*). The mixture was kept at room temperature overnight to prepare a weak gel. The gel was immersed into a strong CaCl${}_{2}$ solution (1 M) for 2 days to finalize the cross-linking process. Figure 1b shows that the proposed method of preparation of alginate gels ensures that samples are transparent and uniform.

Gels of the third series (referred to as Gel-0.1) were prepared using a similar approach. The only difference was that these gels were immersed in 0.1 M CaCl${}_{2}$ solution for 2 h to finalize the gelation process, which made them substantially weaker than the gels of the second series.

After preparation, all gels were immersed into water with pH = 7 overnight to remove unreacted moieties.

### 2.3. Swelling Tests

Equilibrium and transient swelling tests were conducted on alginate gels in aqueous solutions with a pH ranging from 2 to 7 at temperature T=22 °C. To adjust the pH of the solutions, small amounts of 1 M HCl and KOH solutions were added to deionized water. Pre-weighted disc samples of alginate gels (with diameter of 8.5 mm and height of 2.5 mm) were immersed in 1 L aqueous solution with pH = 7, their weights were measured at prescribed times, and the weight changes were recorded as functions of time. After reaching equilibrium at each pH, the pH of the solution was decreased by unity, and the tests were repeated. The degree of swelling *Q* and the equilibrium degree of swelling $Q_{\infty }$ were determined by the formulas

(1) $$\begin{array}{ccc} Q & = & \frac{w_{t}-w_{0}}{w_{0}}, \end{array}$$

(2) $$\begin{array}{ccc} Q_{\infty } & = & \frac{w_{\infty }-w_{0}}{w_{0}}, \end{array}$$

where $w_{0}$, $w_{t}$ and $w_{\infty }$ stand for the weight of a sample after preparation, its weight at time *t*, and the equilibrium weight, respectively.

To characterize changes in the degree of swelling induced by degradation of alginate gels, samples were first immersed in aqueous solutions with pH = 2 for 2 h. Afterwards, they were re-swollen in solutions with various pHs (ranging from 2 to 7) until equilibrium. Experiments on re-swelling were performed at temperatures T=22, 30, 37, and 50 °C that belong to the interval of temperatures relevant to biomedical applications.

To evaluate the influence of ionic strength on the kinetics of re-swelling, as-prepared samples and the samples degraded for 2 h in solution with pH = 2 were immersed in aqueous solutions of NaCl with molar concentrations of NaCl 0.01, 0.1, and 0.2 M, and their degrees of swelling *Q* were monitored as functions of time *t* at room temperature T=22 °C.

All measurements were repeated at least five times on different samples prepared by the same procedure. Experimental data are presented as the mean values. Data scatter is relatively small: The standard deviations of observations in all tests do not exceed 5% of their mean values.

## 3. Results and Discussion

### 3.1. Swelling of Alginate Gels in Solutions with Various pH

**Figure 2:**
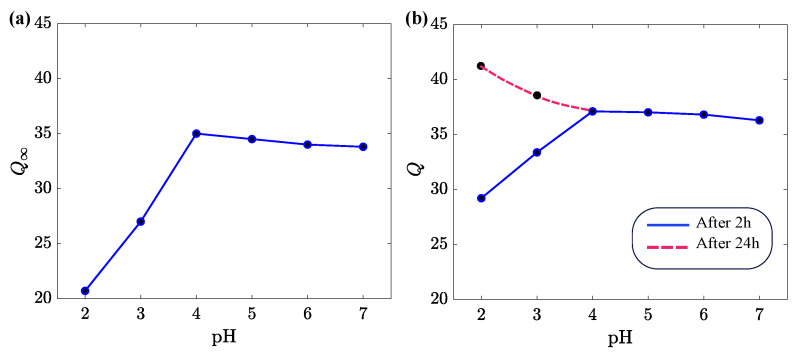
(**a**) Equilibrium degree of swelling $Q_{\infty }$ versus pH for the alginate gel prepared by the conventional method. (**b**) Degree of swelling *Q* versus pH for the uniform alginate gel. Circles: experimental data, lines: guides for eye.

To assess the effect of pH on swelling of alginate gels, their equilibrium degree of swelling $Q_{\infty }$ was measured in aqueous solutions with a pH between 2 and 7. The results are presented in Figure 2.

Samples prepared by the conventional method reached equilibrium after one day of swelling. The swelling diagram in Figure 2a is typical of anionic gels [41,42]. The equilibrium degree of swelling $Q_{\infty }$ increases with pH when pH is below the acid dissociation constant pK${}_{a}$ for alginic acid (its value belongs to the interval between 3.6 for mannuronic acid and 3.8 for guluronic acid [43]) and becomes practically independent of pH when this parameter exceeds pK${}_{a}$. This behavior is explained as follows. Due to the high concentrations of H${}^{+}$ ions in aqueous solutions with low pH, the carboxyl groups (which do not form ionic complexes with Ca${}^{2+}$ ions) become protonated. The protonation (de-ionization) of bound charges leads to a decrease in electrostatic repulsive forces between chains, and, as a consequence, induces shrinkage (de-swelling) of the gel.

The influence of pH on the degree of swelling *Q* of uniform alginate gels is illustrated in Figure 2b. This figure shows that the gels reach equilibrium in a way that differs from that obtained for the conventional inhomogeneous samples. After 2 h of deswelling in solutions with pH below pK${}_{a}$, the samples start to re-swell, and their degree of swelling *Q* (reached after 24 h) exceeds the equilibrium degree of swelling of samples not subjected to degradation at pH = 2. A similar behavior was also observed in tests on Gel-0.1 (Figure S2). After 3 h of degradation of Gel-0.1 in an aqueous solution with pH = 2, samples re-swelled and their degree of swelling exceeded that observed on as-prepared specimens.

The difference between the responses of inhomogeneous and uniform alginate gels may be explained by the aggregation of ionic cross-links (egg-box blocks) in inhomogeneous gels into relatively large clusters [44]. Degradation of the uniform gel (driven by the exchange of Ca${}^{2+}$ and H${}^{+}$ ions and unzipping of chains) causes a noticeable increase in its $Q_{\infty }$ in solutions with a low pH (Figure 2b), while this decrease is prevented by the clustering of ionic cross-links in the conventional inhomogeneous gel (Figure 2a).

### 3.2. The Effects of pH on Shrinkage and Re-Swelling of Alginate Gels

**Figure 3:**
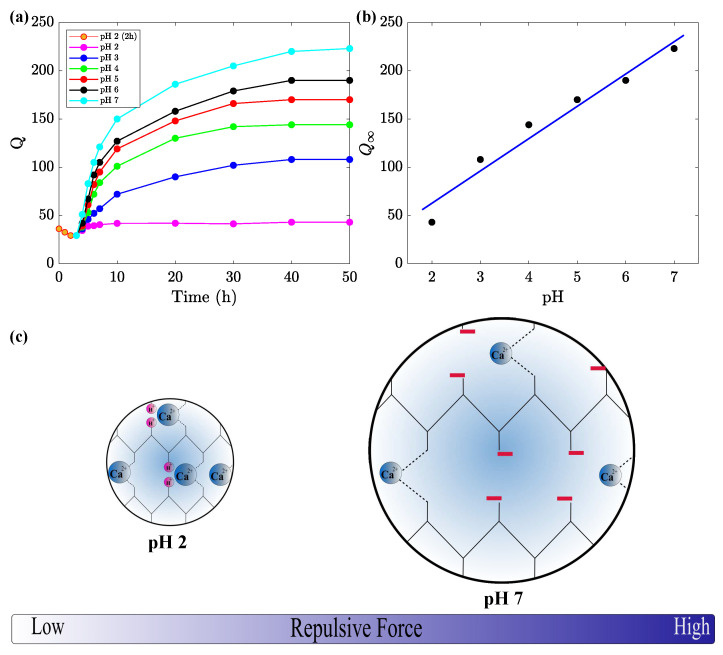
(**a**) Degree of swelling *Q* versus time *t* in aqueous solutions with various pH after immersion of samples in solution with pH = 2 for 2 h. Circles: experimental data, solid lines: guides for eye. (**b**) The equilibrium degree of swelling $Q_{\infty }$ versus pH. Circles: experimental data, solid line: Their approximation by the linear function. (**c**) The effect of pH on repulsive forces between alginate chains.

We begin with an analysis of swelling of uniform alginate gel in an aqueous solution with pH = 2. After immersion into the solution, the gel shrinks and reaches its minimum degree of swelling within 2 h. Afterwards, the gel re-swells and reaches a new equilibrium after about 10 h. The experimental data are presented in Figure 3a, where degree of swelling *Q* is plotted versus the time *t* that elapsed from the start of the experiment. An analogous response of a double-network poly(vinyl alcohol)/sodium alginate gel was previously reported in [45].

After the immersion of alginate gel into a solution with pH = 2, carboxyl groups (belonging mainly to the M blocks) are slowly protonated (de-ionized). This induces shrinkage of the gel at the initial time interval, caused by a decay in the electrostatic repulsive forces. When soaking proceeds, some weak ionic bonds between G and MG blocks break, the corresponding Ca${}^{2+}$ ions are released from the gel, while the carboxyl groups connected by these ions are protonated in the solution with a high concentration of H${}^{+}$ ions. A similar scenario was suggested in [46] to explain another phenomenon.

The deionization of carboxylic groups of the M blocks reduces the degree of swelling, whereas the breakage of ionic bonds between G and MG blocks (mechanical weakening of the gel) causes an increase in this parameter. The exchange of Ca${}^{2+}$ and H${}^{+}$ ions and release of Ca${}^{2+}$ ions from the gel proceed until a new equilibrium is established between elastic forces in the polymer network, electrostatic repulsive forces between bound charges, and ionic and osmotic pressures in the liquid phase of the gel [42].

We proceed with the study of changes in the degree of swelling caused by the immersion of uniform alginate gel into an aqueous solution with a fixed pH after degradation of the gel for 2 h in a solution with pH = 2. Figure 3a shows a significant increase in the equilibrium degree of swelling $Q_{\infty }$ induced by high-electrostatic-repulsive forces between negatively charged carboxyl groups. This conclusion confirms the assumption that, after the breakage of some ionic bonds between alginate chains in the solution with pH = 2, the concentration of carboxyl groups that can be ionized in solution with pH = 7 exceeds their concentration immediately after preparation [45].

The growth of the equilibrium degree of swelling $Q_{\infty }$ of alginate gel, induced by its degradation in a solution with pH = 2, is also observed in aqueous solutions with pH = 3, 4, 5, and 6. When the pH of these solutions decreases, the degree of ionization of bound charges is reduced. As a consequence, repulsive forces between polymer chains weaken, and the equilibrium degree of swelling decreases. The dependence of $Q_{\infty }$ on the pH of aqueous solutions in which re-swelling is conducted is reported in Figure 3b. The data in this figure are approximated by the linear function.

Similar observations on Gel-0.1 in aqueous solutions with various pHs (driven by 3 h of degradation in a solution with pH = 2) are reported in Figure S3. To evaluate the accuracy of the above assumptions, alginate samples were immersed for 2 h in an aqueous solution with pH = 3 (this value is rather close to pK${}_{a}$) and re-swollen in a solution with pH = 7. The increase in the equilibrium degree of swelling observed under re-swelling correlates reasonably well with the dependence reported in Figure 3b (data are not presented for brevity).

### 3.3. The Effect of Ionic Strength on Swelling of Alginate Gels

**Figure 4:**
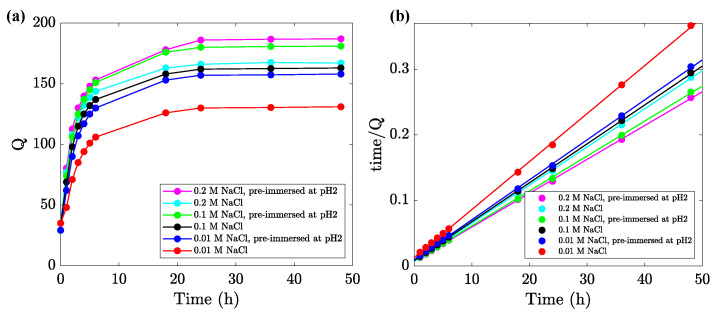
(**a**) Degree of swelling *Q* versus time *t* in aqueous solutions of NaCl. Circles: experimental data, solid lines: guides for eye. (**b**) Approximation of the experimental data by Equation (3).

To analyze the influence of ionic strength of aqueous solution on swelling of uniform alginate gel, samples were degraded by soaking for 2 h in a solution with pH = 2. Then, they were immersed in solutions with pH = 7 and various molar concentrations (0.01, 0.1, and 0.2 M) of NaCl, and their degrees of swelling *Q* were measured as functions of time *t* elapsed after immersion. Experimental data in the re-swelling tests are reported in Figure 4a (and Figure S4a for Gel-0.1), where *Q* is plotted versus *t*. For comparison, observations on as-prepared alginate gels (not soaked in solution with pH = 2) are also presented in these figures.

Figure 4a and Figure S4a show a pronounced growth in the degree of swelling *Q* with time *t* under re-swelling. This increase is caused by the exchange between Ca${}^{2+}$ and H${}^{+}$ ions [47,48], coupled with unzipping reactions (breakage of ionic bonds between chains) [44]. The unzipping reactions are driven by forces that are transmitted from one egg-box block to adjacent blocks, inducing the progressive cleavage and uncross-linking of the gel [49].

It is natural to presume that the weaker a gel is, the more intensive the exchange that occurs between Ca${}^{2+}$ and H${}^{+}$ ions, and the more pronounced the unzipping of ionic bonds between alginate chains. This assertion is confirmed by experimental data on Gel-0.1, which were fully unzipped in 0.1 M NaCl solution and demonstrated a strong increase over time in their degree of swelling after immersion in 0.01 M NaCl solutions (Figure S4a).

An interesting and unexpected feature of the data reported in Figure 4a is that the equilibrium degree of swelling $Q_{\infty }$ increases monotonically with the ionic strength of aqueous solutions. Conventional anionic polyelectrolyte gels reveal a decay in $Q_{\infty }$ with ionic strength. This decrease is explained by the reduction in repulsive forces between chains, driven by the formation of ionic complexes between negatively charged bound groups and positively charged mobile Na${}^{+}$ ions [50,51]. The same response was observed in equilibrium swelling tests on alginate gels prepared by the standard procedure [52] and wet-spin alginate fibers [53]. On the contrary, Figure 4a and Figure S4a reveal a pronounced growth of $Q_{\infty }$ with molar fractions of NaCl. This growth may be explained by the fact that alginate chains in a polymer network are bridged by ionic and hydrogen bonds [54], and Na${}^{+}$ ions serve as bond-breaking agents for weak intermolecular interactions (see review [55] for a discussion). The effect of mobile cations in aqueous solutions on the strength of hydrogen bonds between alginate chains remains an unresolved problem that requires further investigation [56,57].

To study the kinetics of swelling of alginate gel in solutions of NaCl, the experimental data in Figure 4a and Figure S4a were replotted in Figure 4b and Figure S4b. Each set of data was approximated separately by the pseudo-second-order model

(3) $$\frac{t}{Q}=\frac{1}{kQ_{\infty }^{2}}+\frac{t}{Q_{\infty }}.$$

An advantage of this relation is that it involves the swelling rate *k* as the only adjustable parameter. Equation (3) was proposed in [58] to describe the adsorption kinetics and applied to the analysis of swelling of hydrogels in [59]. Figure 4b and Figure S4b show that the model (3) adequately describes the swelling kinetics of the alginate gel under consideration in NaCl solutions. The coefficient *k* increases with NaCl concentration for non-degraded samples (from $k=11.7\times 10^{-7}$ to $k=13.9\times 10^{-7}$ s${}^{-1}$ when the molar fraction of NaCl grows from 0.01 M to 0.2 M) and decreases for degraded specimens (from $k=11.0\times 10^{-7}$ to $k=9.8\times 10^{-7}$ s${}^{-1}$ under the same conditions).

### 3.4. The Effect of Temperature on Swelling of Alginate Gels

**Figure 5:**
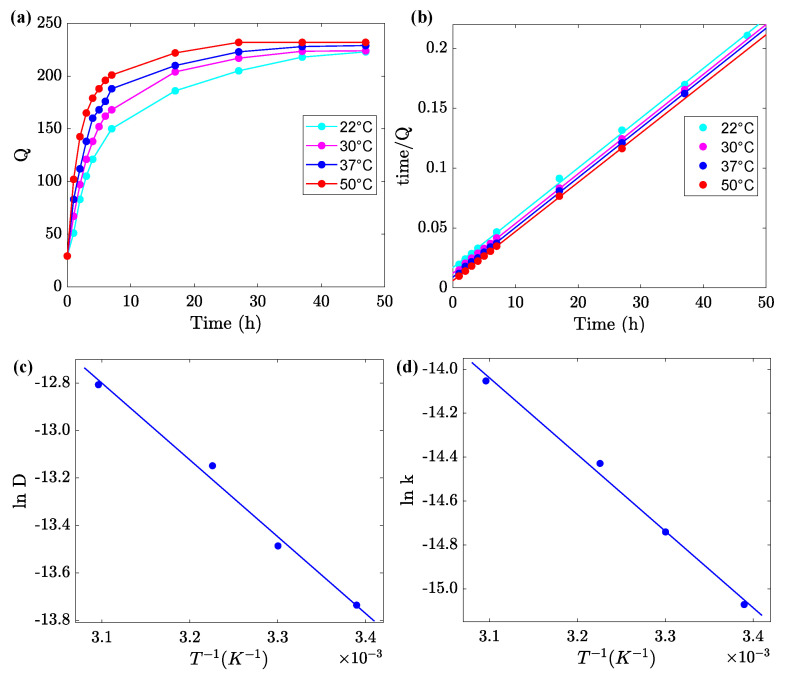
(**a**) Degree of swelling *Q* versus time *t* at various temperatures *T*. Circles: experimental data, solid lines: guides for eye. (**b**) Fitting of the data by Equation (3). (**c**,**d**) Coefficients *D* and *k* versus temperature *T*. Circles: treatment of observations, solid lines: approximation of the data by Equation (5).

To assess the effect of temperature *T* on the swelling of uniform alginate gel, samples were immersed for 2 h in an aqueous solution with pH = 2 at room temperature and transmitted to a solution with pH = 7 at temperature *T*. Changes in their degree of swelling *Q* with time *t* elapsed after transmission were monitored. Tests were performed at temperatures T=22, 30, 37 and 50 °C. Experimental data are reported in Figure 5a, where *Q* is plotted versus time *t*. Observations on Gel-0.1 in a series of similar tests at temperatures T=22, 28, 32, 37 and 50 °C are presented in Figure S5a. A comparison of these figures shows that the equilibrium degree of swelling $Q_{\infty }$ is practically independent of temperature *T*, but it is strongly affected by the molar fraction of CaCl${}_{2}$ used in the preparation procedure.

The kinetics of re-swelling of the gels are characterized by the coefficient of diffusion of water *D*. This parameter was calculated by fitting initial intervals of the experimental dependencies Q(t) using Equation [60,61]

(4) $$\frac{Q}{Q_{\infty }}=\frac{4}{a}{\left(\frac{Dt}{\pi }\right)}^{\frac{1}{2}},$$

where *a* denotes height of a disk sample. Each set of data in Figure 5a and Figure S5a was matched separately (only observations obeying the condition $Q/Q_{\infty }<0.6$ were approximated), and the best-fit values of *D* were determined. An advantage of Equation (4) is that it involves the only material parameter *D*. Its shortcoming is that this relation presumes *D* to be independent of degree of swelling *Q*, which contradicts numerous observations [62]. More sophisticated models accounting for the influence of swelling on diffusivity of solvents in hydrogels were developed in [63,64].

To assess the accuracy of calculations, each set of data in Figure 5a and Figure S5a was replotted in Figure 5b and Figure S5b and fitted by Equation (3) with the only adjustable parameter *k*. The effect of temperature *T* on the coefficients *D* and *k* is described by the Arrhenius equations

(5) $$D=D_{0}\exp\left(-\frac{E_{a}}{RT}\right),\;k=k_{0}\exp\left(-\frac{E_{a}}{RT}\right),$$

where $D_{0}$ and $k_{0}$ stand for pre-factors, $E_{a}$ is the activation energy for diffusion of solvent, *R* is the universal gas constant, and *T* denotes the absolute temperature.

Approximation of the data in Figure 5c,d by Equation (5) implies that $E_{a}=26.9$ kJ/mol and $E_{a}=29.0$ for the coefficients *D* and *k*, respectively. The closeness of these values may serve as a confirmation of the accuracy of our analysis.

## 4. Conclusions

Homogeneous alginate gels cross-linked by Ca${}^{2+}$ ions were prepared by using a novel two-step procedure, and their swelling properties were studied experimentally.

Our analysis of swelling diagrams on uniform alginate gels in aqueous solutions with various pHs demonstrates that their equilibrium degree of swelling $Q_{\infty }$ adopts similar values to those of inhomogeneous alginate gels prepared by the conventional procedure when pH exceeds the acid dissociation constant pK${}_{a}$ for alginic acid. However, the swelling of uniform gels in aqueous solutions with a low pH (below pK${}_{a}$) reveals a different behavior. After the immersion of a sample into a solution with a low pH, the sample shrinks at the initial interval of time and re-swells afterwards due to the breakage of ionic bonds between chains (treated as degradation of the gel). The equilibrium degree of swelling of the uniform gel exceeds that of the inhomogeneous gel prepared by the conventional method (Figure 2).

The work focuses on the study of equilibrium and transient swelling of alginate gels induced by the cleavage of ionic bonds in aqueous solutions with a low pH. This shows that this degradation (caused by the exchange of Ca${}^{2+}$ and H${}^{+}$ ions and partial unzipping of egg-box ionic bonds) makes alginate gels sensitive to the environmental conditions (pH, ionic strength and temperature of aqueous solutions), as well as to the loading history. As sensitivity to the environmental stimuli is a characteristic feature of the polymer networks connected by catch bonds, we suppose that alginate gels may serve as a simple model, mimicking the behavior of more sophisticated structures in living matter.

It is revealed that, after the partial degradation of uniform alginate gels in solution with pH = 2 and the immersion of samples in aqueous solutions with a higher pH, their equilibrium degree of swelling *Q* noticeably increases, in proportion to the pH of the solution at which re-swelling occurs (Figure 3a,b).

Unlike inhomogeneous alginate gels prepared by the conventional procedure, whose behavior resembles that of anionic polyelectrolyte gels, uniform gels immersed into NaCl solutions with pH = 7 (after degradation in aqueous solution with pH = 2) have an unusual response. Their equilibrium degree of swelling $Q_{\infty }$ noticeably increases with the ionic strength of the solutions (Figure 4a). This may be explained by the breakage of hydrogen bonds between alginate chains, driven by the presence of Na^+^ cations acting as bond-breaking agents.

The re-swelling rate of uniform gels in solutions with pH = 7 (after their degradation in solution with pH = 2) strongly increases with temperature *T* (ranging 20 to 50 °C). The increase in the solvent diffusivity is governed by the Arrhenius dependence with an activation energy $E_{a}$ close to 30 kJ/mol, which is the characteristic value for conventional alginate gels (Figure 5).

This study demonstrates that uniform alginate gels prepared by the proposed method preserve their integrity in aqueous solutions with a pH ranging from 2 to 7 and ionic strength in the interval from 0 to 0.2 M at temperatures ranging from room temperature up to 50 °C, and can be used in biomedical applications.

## Data Availability

The data that support the findings of this study are available from the corresponding author, ADD, upon reasonable request.

## Supplementary Materials

The following supporting information can be downloaded at: https://www.mdpi.com/article/10.3390/ijms24065064/s1.
