# Supplementary material for: Swelling of Homogeneous Alginate Gels with Multi-Stimuli Sensitivity
Source: Int J Mol Sci. 2023 Mar 7;24(6):5064. doi: 10.3390/ijms24065064 (PMC10049665; doi:10.3390/ijms24065064)
Supplement: Supplementary file 1 [file ijms-24-05064-s001.zip › ijms-2248409-supplementary.pdf]

Supplementary material
Mechano-sensitive alginate gels with multi-stimuli-responsive swelling

H. Malektaj, A.D. Drozdov, J. deClaville Christiansen

Department of Materials and Production

Aalborg University

Fibigerstraede 16, Aalborg 9220, Denmark

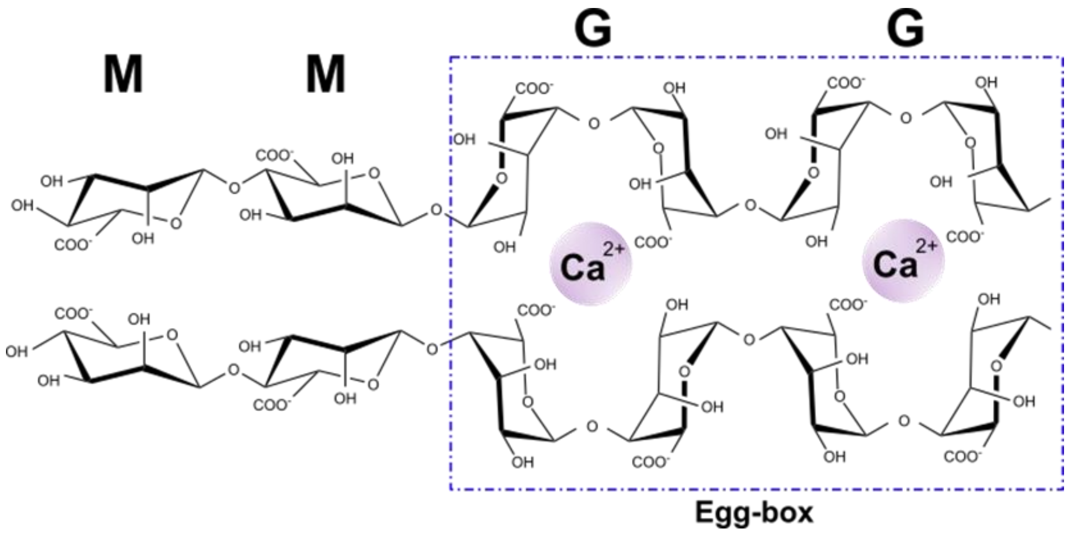

Figure S1. Molecular structure of alginate chains consisting of G and M blocks, and the egg-box model.

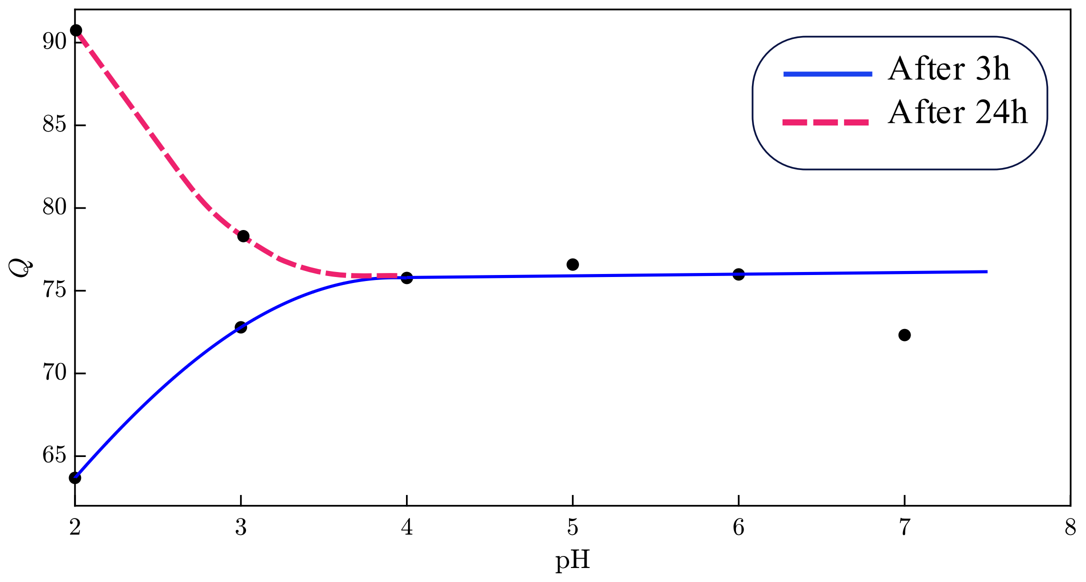

Figure S2. Degree of swelling *Q* of Gel-0.1 versus pH.

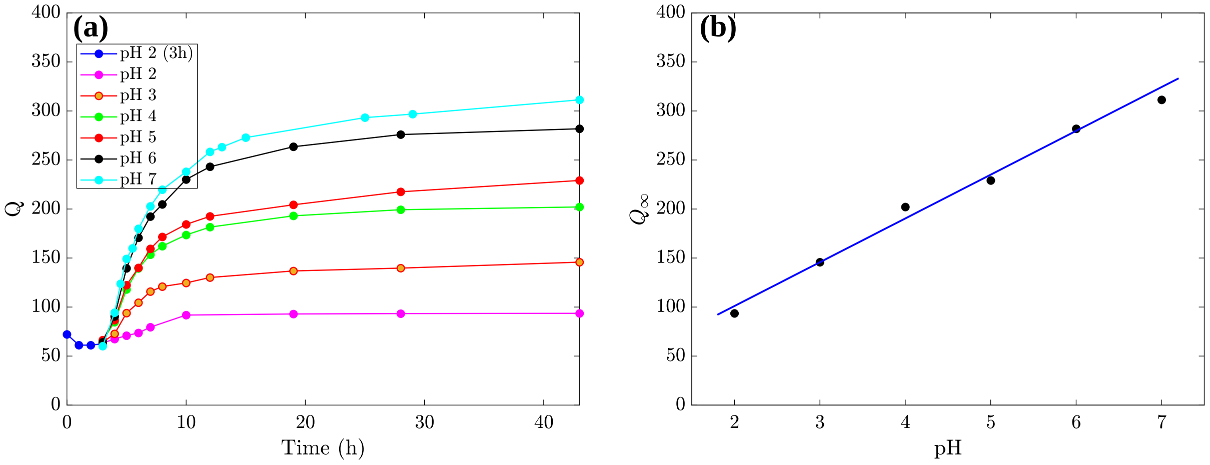

Figure S3. a) Degree of swelling Q versus time t for Gel-0.1 in aqueous solutions with various pH after immersion of the gel in solution with pH=2 for 3h. b) The equilibrium degree of swelling Q_∞ of Gel-0.1 versus pH.

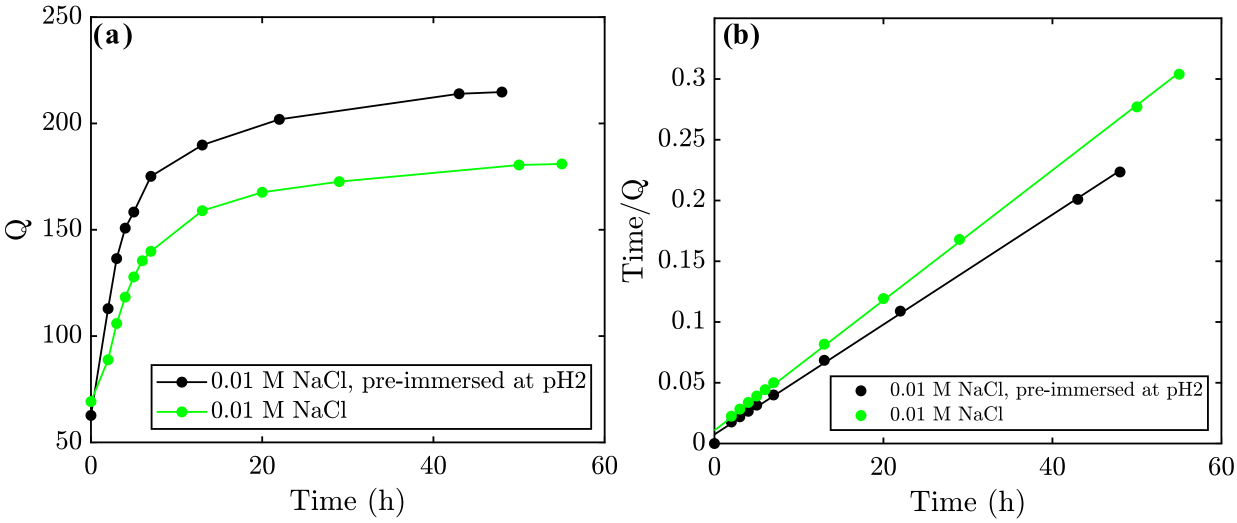

Figure S4. a) Degree of swelling *Q* of Gel-0.1 versus time *t* in aqueous solution with 0.01M NaCl. b) Fitting of the data for Gel-0.1 by Eq. 3.

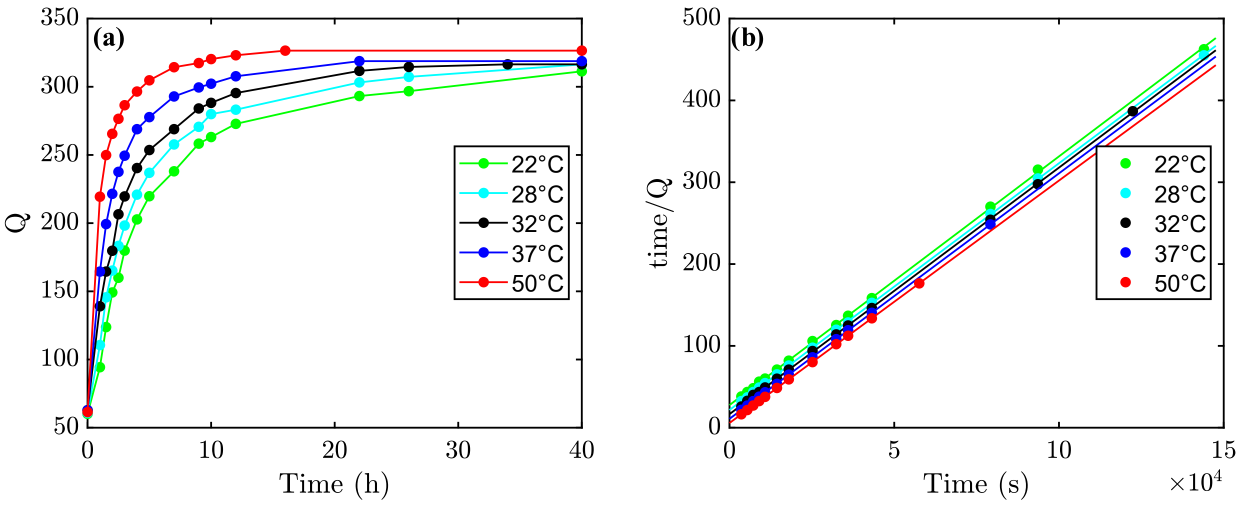

Figure S5. a) Degree of swelling *Q* of Gel-0.1 versus time *t* at various temperatures *T*. b) Fitting of the data for Gel-0.1 by Equation (3).
